# Measuring single‐cell immune clonality to track haematological cancers

**DOI:** 10.1002/ctm2.1780

**Published:** 2024-08-20

**Authors:** Amos Choo, Zewen Kelvin Tuong

**Affiliations:** ^1^ Ian Frazer Centre for Children's Immunotherapy Research, Child Health Research Centre, Faculty of Medicine The University of Queensland Brisbane Australia

**Keywords:** blood cancer, clonality, paediatric, Single‐cell, T‐cell receptor

## Abstract

While paediatric blood cancers are deadly, modern medical advances have enabled clinicians to measure levels of residual cancer cells to manage therapeutic strategies for patients. However, blood cancers, including leukaemias and lymphomas, are highly heterogeneous and is comprised of complex clonal populations that can hinder efforts in detecting the cancer cells as well as managing treatments. Furthermore, the tumour microenvironment is comprised of heterogenous immune dynamics that may be different between patients. High‐throughput sequencing has constributed to new discoveries in genetic and transcriptomic alterations underpinning cancer, including blood cancers, and has changed how patients are monitored and managed. Here we discuss the recent efforts using single‐cell approach, particularly on efforts to track clonal heterogenity of paediatric blood cancer and the underlying immune response, highlighting avenues for novel biomarker discovery that may have significant impact on clinical oncology practice.

Diagnosing and treating leukaemia and lymphomas remains a significant challenge. These malignancies often induce heterogeneity in the immune response, causing disease management complications. Recent advancements in sequencing technologies have shifted to single‐cell sequencing methods such as single‐cell RNA‐sequencing (scRNA‐seq) and single‐cell immune repertoire sequencing of the B‐cell receptors (BCR; scBCR‐seq) or T‐cell receptor (TCRs; scTCR‐seq).^1^ These techniques enable a more precise dissection of the immune cell development and immune response at a high resolution, revealing the intrinsic details of adaptive immune cell types involved, including the BCR/TCR clonality patterns. Therefore, they hold incredible potential for understanding the immune mechanisms underpinning cancer development and response to treatment.

In the context of haematological malignancies, scRNA‐seq and scTCR‐seq have been useful in tracing immune and cancer cell lineages and understanding disease heterogeneity. For example, separate analysis involving phylogenetic tracing of BCR lineages with scBCR‐seq, and pseudotime trajectory analysis with scRNA‐seq, helped discover how tumour sites in follicular B‐cell lymphoma evolved distinctly.^2^ Similarly, scTCR‐seq enabled the decoding of the clonal architecture in diseases like T‐acute lymphoblastic leukaemia (T‐ALL) and acute myeloid leukaemia (AML), providing valuable insights into clonal expansions and antigen‐driven selection process. In particular, in a study conducted by Abbas et al., they used a single‐cell gene expression and immune profiling approach to examine the bone marrow cells in patients with relapsed AML and found that the responders to programmed cell death 1 blockade therapy exhibited expansion of CD8+ T‐cell clonotypes, indicating an active adaptive immune response induced by the checkpoint inhibitor drug, whereas treatment‐resistant patients exhibited a contraction of their TCR repertoire.^3^ Using pseudotime trajectory analysis, they also uncovered a population of CD8+ T cell phenotypes expressed stem‐like properties, providing potential for long‐term immune memory and sustained response to therapy.^3^ Similarly, a separate study by Wu et al. performed a similar analysis in normal karyotype AML (NK‐AML) patients, discovering eight distinct T‐cell clusters which represented the different subpopulations of T cells within the NK‐AML context.^4^ Furthermore, specific T‐cell clusters were shown to contain marked clonal expansion in those patients, suggesting that these clusters play an important role in the immune response within the tumour microenvironment.^4^ scRNA‐seq integrated with scTCR profiling was also used to infer pathophysiologic mechanisms of clonal expansion and persistence of T cells in large granular lymphocyte leukaemia (T‐LGLL), including the discovery of markedly reduced TCR repertoire diversity and enrichment of immunosuppressive ‘exhausted’ T cell signatures in the T‐LGLL patients.^5^

Identifying biomarkers through scTCR‐seq is attractive because it offers the ability to resolve how the immune system responds to cancer progression or treatment through detailed analysis of the T cell repertoire and gene expression at a single cell level. For example, one could identify specific TCR sequences associated with effective anti‐tumour response in a patient's immune repertoire, thereby serving as a robust biomarker for cancer diagnosis and personalised immune therapies. In particular, DeepTCR introduced a deep‐learning framework that incorporates information found in scTCR‐seq data, including the V/D/J gene usage and CDR3 sequence information. This framework would then provide a singular interface for other users to perform various downstream tasks such as paired α/β chain analysis, visualisation and clustering. Zhu went a step further and implemented scNAT, a deep learning method to integrate scRNA‐seq and scTCR‐seq data as a unified representation data in a latent space for downstream analysis.^6^ They demonstrated that scNAT can more robustly identify cell clusters and infer T cell trajectory.^6^ A main significant advantage scNAT offers over DeepTCR is the addition of transcriptome data into the analysis, resulting in more precise cell clustering.

One avenue where paired single‐cell gene expression and immune repertoire analysis may also particularly be useful is in lineage tracing of haematological cancer clones through their immune receptors, for instance in mixed lineage ALL where the leukaemia cells can display clonal rearrangements of TCR and BCRs.^7^ Mixed lineage ALLs, while rare, are considered high‐risk ALLs and represent a clinical conundrum. Patients with mixed lineage ALLs generally have poorer treatment response and outcomes.^8^ Trajectory analysis for these types of haematological cancers may be particularly informative on the progression and evolution of the disease. Computational workflows to define the overall TCR trajectories have been recently developed, including one by Xie et al.^9^ where a pseudotime trajectory is constructed based on the T‐cell gene expression information and the trajectory is then subsequently mapped with the meaningful clonotype clusters. For example, in acute lymphocytic choriomeningitis virus infection, Xie used their tool to show that some subclones have distinct biases differentiation associated with disease staging and prognosis. Perhaps particularly useful in the context of tracing mixed ALL, Suo et al. developed Dandelion to model cell‐fate trajectories of developing T‐cells directly using the TCR rearrangements through a (gene expression‐based) pseudobulking approach and accurately aligned CD4/CD8 T‐cell fate commitment at both the gene expression and TCR level.^10^ We envisage that this workflow can be used to project the mixed lineage ALL TCR/BCR configurations onto a normal TCR/BCR trajectory, allowing researchers to map the root cell states associated with the leukaemia of ambiguous lineage, which can potentially shed light on new biomarkers and/or new therapeutic strategies.

In conclusion, clinical oncology can benefit from single‐cell gene expression and immune repertoire analysis to understand the intricate details of individual cell types in the immune response to disease progression and treatment resistance (Figure 1). While these technologies still have not been implemented clinically, they are increasingly adopted in clinical trials; they hold considerable potential as the findings can be actioned to develop novel biomarkers and aid in the clinical management of patients with cancer.

**FIGURE 1:**
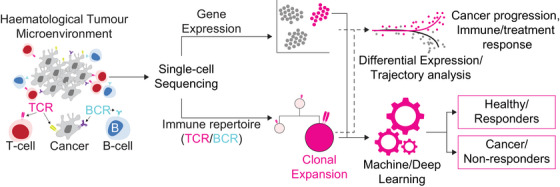
Illustration of the utility of combined single‐cell profiling of the gene expression and immune repertoire landscape. Pairing the two technologies can help to better understand cancer progression, immune response and treatment outcomes, as well as to identify unique biomarkers for patients. This is because, on top of the underlying transcriptome programming, adaptive immune cells, including T‐ and B‐cells, and some subtypes of haematological cancers will express the adaptive immune receptors, allowing clinicians and researchers to track the individual clones for prognostication purposes.

## AUTHOR CONTRIBUTIONS

Amos Choo and Zewen Kelvin Tuong conceived, designed, wrote and edited the manuscript. Zewen Kelvin Tuong prepared figures. Zewen Kelvin Tuong supervised the work.

## CONFLICT OF INTEREST STATEMENT

Zewen Kelvin Tuong has consulted for Synteny Biotechnology in the last 3 years. Amos Choo declares no conflict of interest.

## ETHICS STATEMENT

This manuscript does not report on or involve the use of any animal or human data or tissue.

## References

[1] Irac SE , Soon MSF , Borcherding N , Tuong ZK . Single‐cell immune repertoire analysis. Nat Methods. 2024;21(5):777‐792. doi:10.1038/s41592-024-02243-4 38637691

[2] Haebe S , Shree T , Sathe A , et al. Single‐cell analysis can define distinct evolution of tumor sites in follicular lymphoma. Blood. 2021;137(21):2869‐2880. doi:10.1182/blood.2020009855 33728464 PMC8160505

[3] Abbas HA , Hao D , Tomczak K , et al. Single cell T cell landscape and T cell receptor repertoire profiling of AML in context of PD‐1 blockade therapy. Nat Commun. 2021;12(1):6071. doi:10.1038/s41467-021-26282-z 34663807 PMC8524723

[4] Wu W , Liang X , Li H , et al. Landscape of T cells in NK‐AML(M4/M5) revealed by single‐cell sequencing. J Leukoc Biol. 2022;112(4):745‐758. doi:10.1002/JLB.5A0721-396RR 35258858

[5] Gao S , Wu Z , Arnold B , et al. Single‐cell RNA sequencing coupled to TCR profiling of large granular lymphocyte leukemia T cells. Nat Commun. 2022;13(1):1982. doi:10.1038/s41467-022-29175-x 35411048 PMC9001664

[6] Zhu B , Wang Y , Ku LT , et al. scNAT: a deep learning method for integrating paired single‐cell RNA and T cell receptor sequencing profiles. Genome Biol. 2023;24(1):292. doi:10.1186/s13059-023-03129-y 38111007 PMC10726524

[7] Mi X , Griffin G , Lee W , et al. Genomic and clinical characterization of B/T mixed phenotype acute leukemia reveals recurrent features and T‐ALL like mutations. Am J Hematol. 2018;93(11):1358‐1367. doi:10.1002/ajh.25256 30117174 PMC8193761

[8] Lao ZT , Ding LW , An O , et al. Mutational and transcriptomic profiling of acute leukemia of ambiguous lineage reveals obscure but clinically important lineage bias. Haematologica. 2019;104(5):e200‐e203. doi:10.3324/haematol.2018.202911 30514800 PMC6518916

[9] Xie J , Jeon H , Xin G , Ma Q , Chung D . LRT: integrative analysis of scRNA‐seq and scTCR‐seq data to investigate clonal differentiation heterogeneity. PLOS Comp Biol. 2023;19(7):e1011300. doi:10.1371/journal.pcbi.1011300 PMC10358952 37428794

[10] Suo C , Polanski K , Dann E , et al. Dandelion uses the single‐cell adaptive immune receptor repertoire to explore lymphocyte developmental origins. Nat Biotechnol. 2024;42(1):40‐51. doi:10.1038/s41587-023-01734-7 37055623 PMC10791579

